# Case Report: Castleman Disease With an Associated Stromal Spindle Cell Proliferation, *PDGFRB* Mutation and p53 Expression: Clonal Origins of a Rare Disease

**DOI:** 10.3389/fonc.2022.857606

**Published:** 2022-04-13

**Authors:** Kunwar I. Singh, Sumanth Gollapudi, Jyoti Kumar, Alexandra Butzmann, Corinn Small, Sara Kreimer, Emine Arzu Saglam, Roger Warnke, Oscar Silva, Robert S. Ohgami

**Affiliations:** ^1^ Department of Pathology, University of California, San Francisco, San Francisco, CA, United States; ^2^ Department of Pathology, Stanford University, Stanford, CA, United States

**Keywords:** Castleman disease, PDGFRB, TP53, p53, fibroblastic reticular cell, follicular dendritic cell, FRC, FDC

## Abstract

Castleman disease (CD) is a rare lymphoproliferative disorder with distinct clinical subtypes. However, our understanding of the underlying pathogenesis of particular subtypes of CD remains unclear. While the characteristic morphologic changes within UCD, including occasional cases of overgrowth of spindled stromal and follicular dendritic cells have been described, the nature and origin of these spindle cells remain elusive. Few reports have suggested that underlying stromal cells in UCD are clonally neoplastic and may be of fibroblastic reticular cell (FRC) or follicular dendritic cell (FDC) origins given their close clonal relationship. Although certain histomorphologic features may aid diagnosis, there are no specific biomarkers that can differentiate a reactive process mimicking UCD from true UCD. Hence, we describe an index case with morphology consistent with the hyaline vascular subtype of UCD with concomitant atypical smooth muscle actin (SMA)-positive stromal spindle cell proliferation containing a recurrent PDGFRB N666S mutation and upregulation of p53 expression. Further analysis of 21 additional cases of UCD identified increased p53 expression by digital image analysis and SMA positive stromal cells predominantly within the paracortical and intrafollicular areas further strengthening the hypothesis of the stromal cellular derivation and origins of UCD.

## Introduction

Castleman disease (CD) is a rare lymphoproliferative disorder that can be classified into two distinct clinical subtypes based on the distribution of lymphadenopathy: unicentric Castleman disease (UCD), inferring a single site of nodal involvement, and multicentric Castleman disease (MCD), involving multiple sites of lymphadenopathy (1, 2). The initial designation of the disease by Benjamin Castleman in 1954 described the histologic features as “angiofollicular lymph node hyperplasia” or “giant lymph node hyperplasia” (3). Current classification includes morphologic features (hyaline vascular, mixed, and plasma cell variants), anatomic distribution, and further diagnostic subtypes of MCD (idiopathic, HHV8-associated, and POEMS associated), which impact prognosis, clinical monitoring, and therapeutic strategy (4, 5). However, our understanding of the underlying pathogenesis of particular subtypes of CD remains unclear (6).

While the characteristic morphologic changes within UCD, including occasional cases of overgrowth of spindled stromal and follicular dendritic cells have been described (7), the nature and origin of these spindle cells remain elusive. Few reports have suggested that underlying stromal cells in UCD are clonally neoplastic and may be of fibroblastic reticular cell (FRC) or follicular dendritic cell (FDC) origins given their close clonal relationship (8–10). Furthermore, although certain histomorphologic features may aid diagnosis, there are no specific biomarkers that can differentiate a reactive process mimicking UCD from true UCD.

Here we describe an index case with morphology consistent with the hyaline vascular subtype of UCD with concomitant atypical smooth muscle actin (SMA)-positive stromal spindle cell proliferation containing a recurrent *PDGFRB* N666S mutation and upregulation of p53 expression. An analysis of 21 additional cases of UCD identified increased p53 expression and SMA positive stromal cells predominantly within the paracortical and intrafollicular areas further strengthening the hypothesis of the stromal cellular derivation and origins of UCD.

## Case Description

**Figure 1:**
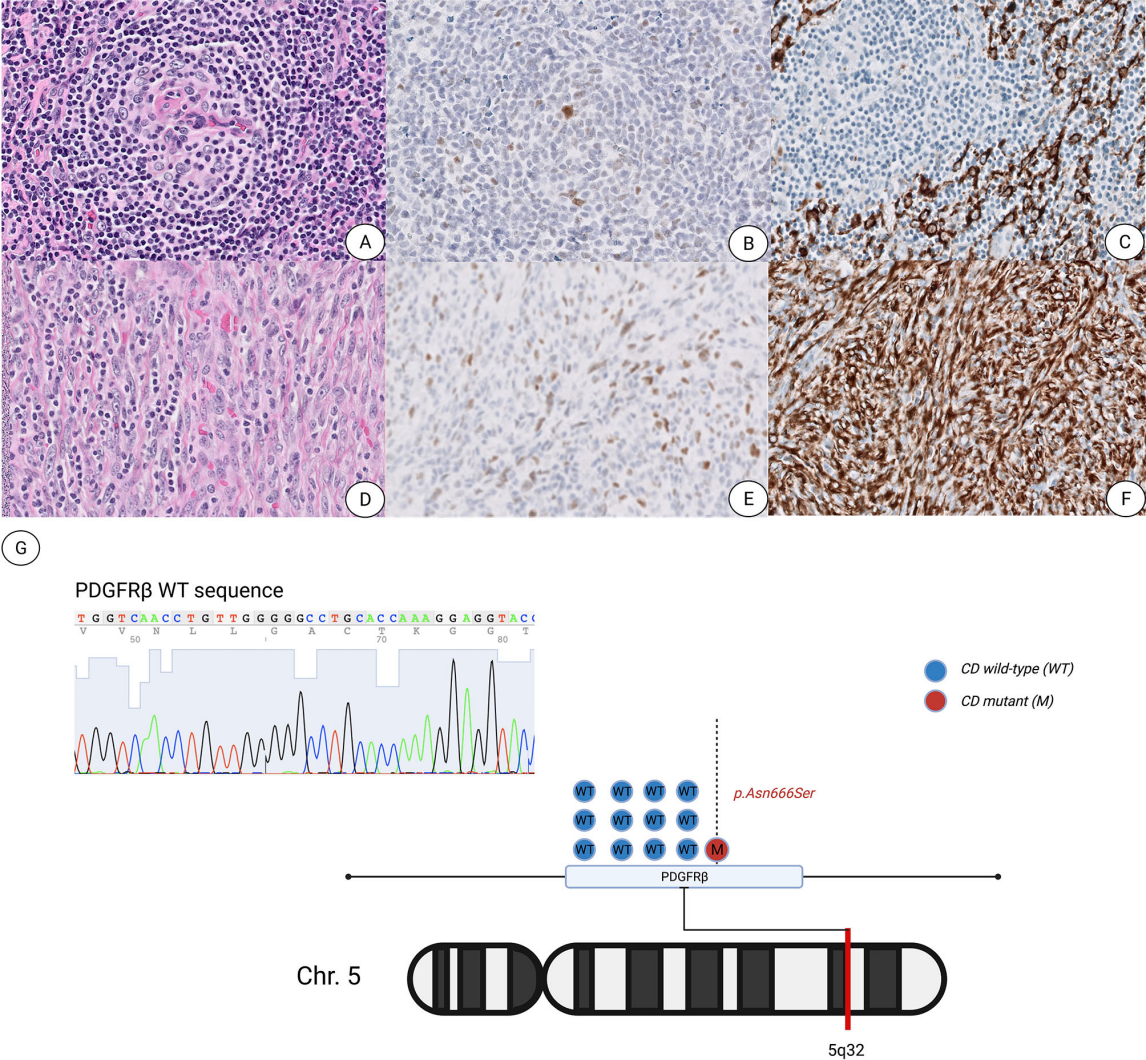
Unicentric Castleman Disease with an associated atypical spindle cell proliferation shows increased p53 protein expression and a *PDGFRB* p.Asn666Ser mutation. **(A–C)** Representative images of characteristic atretic follicle showing hyalinization of the germinal center with a penetrating vessel stained with hematoxylin and eosin **(A)**, p53 **(B)** and SMA **(C)**. **(D–F)** Representative images of spindle cell proliferation stained with H&E **(D)**, p53 **(E)** and SMA **(F)**. All images are presented at ×400 magnification. **(G)** Graphic illustration of the PDGFRB p.Asn666Ser point mutation located within the 5q32 arm of chromosome 5.

A 19-year-old woman was found to have a left subpectoral mass impinging on her brachial plexus. Her initial biopsy revealed morphology consistent with Castleman disease, hyaline vascular variant along with an associated stromal cell proliferation. Due to the persistence of the mass and worsening clinical symptoms, the mass was excised, which revealed similar histomorphology as seen in the prior biopsy (**Figure 1**). Immunohistochemical stains demonstrated that the stromal cell proliferation expressed SMA and caldesmon. However, this proliferation lacked desmin, epithelial membrane antigen, anaplastic lymphoma kinase expression, and FDC markers, CD21, CD23, and somatostatin receptor 2A (SSTR2A). In total, the phenotype of the spindle cells overlapped with the phenotype of fibroblastic reticular cells and/or angiomyoid cells.

Interestingly, p53 overexpression by immunohistochemistry was noted in the spindle cell proliferation, rare FDCs and scattered spindle cells within the areas showing typical CD features (**Figure 1**). Targeted next-generation sequencing (NGS) was performed using a clinically validated panel and identified a *PDGFRB* c.1997 A>C (p.N666S) mutation with a variant allele frequency (VAF) of 6%. Enrichment of DNA from the bland spindle cell proliferation by selective microdissection of the area richest in spindle cells followed by NGS sequencing revealed increased allele frequency of the *PDGFRB* mutation (VAF of 17%). We noted numerous small lymphocytes infiltrating the spindle cell proliferation, comprising 73.5% of the cellular composition in the richest spindle cell area, while the spindle cells constituted approximately 25% of the overall cellularity within the targeted enrichment area harboring the *PDGFRB* mutation with a VAF of 17%. We also observed that the spindle cell proliferation was estimated at 5–10% of the whole tissue section, which correlates as a proportional fraction based on the VAF of 6% as enumerated by NGS performed on the total tissue.

**Figure 2:**
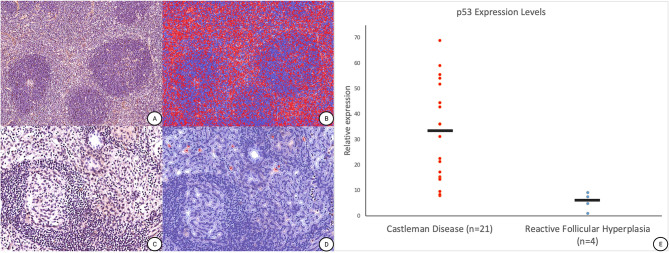
p53 nuclear protein positive cell detection in Unicentric Castleman Disease. **(A)** Representative p53 immunohistochemical stain performed on additional UCD cases (magnification ×100). **(B)** Post cell detection image from QuPath illustrating the presence of p53 nuclear protein overexpression[red] versus background negative cells [blue] (magnification ×100). **(C)** Representative p53 immunohistochemical stain performed on an internal “control” case of reactive hyperplasia (magnification ×200). **(D)** Post cell detection image from QuPath illustrating the presence of p53 nuclear protein overexpression[red] versus background negative cells [blue] (magnification ×200). **(E)** Dot plot of p53 nuclear expression levels within UCD normalized to cases of reactive follicular hyperplasia. Of note, prospective clinical analysis showed that an additional 15 cases of reactive lymph nodes displayed a relative expression of 10 or less on p53 on staining (data not shown).

Based on these findings, an analysis of 21 additional cases of UCD was performed to assess the presence of SMA positive spindle cells with p53 expression. Overall, p53 showed significant overexpression in 18 cases of UCD predominantly within an intrafollicular and paracortical distribution (**Figure 2**) and appeared to colocalize with SMA positive single and grouped cells within the paracortex that were separate from vasculature (**Supplementary Figure 1**).

## Methods

Cases of UCD were identified by retrospective search of the UCSF Department of Pathology archives over the last 20 years (2001–2021). Twenty-one cases were identified and reviewed for diagnostic confirmation (KS and RO). Immunohistochemical staining for p53 and SMA was performed on 5-µm sections with commercially purchased p53 (clone DO-7, Biolegend, San Diego, CA, USA) and SMA (clone 1A4, Invitrogen, Waltham, Massachusetts, USA) antibodies using an antibody dilution of 1:150. Whole slide imaging was performed using an Aperio AT2 scanner (Leica Biosystems, Nussloch, Germany) at ×40 magnification (0.25 µm per pixel).

Digital image analysis (DIA) was performed using a custom pipeline *via* QuPath v0.3.0 (Belfast, UK) (11, 12). Parameters for cell detection were optimized using staining vector estimation (hematoxylin, 3,3’-diaminobenzidine antibody, and residual) for each tissue sample. A single staining intensity threshold was used for the assessment of cell detection. Heatmaps were then manually analyzed to assess for distribution of staining. The internal “control” samples were concomitant benign regional lymph nodes excised in a subset of patients with UCD and used to normalize positive expression levels by assessing background non-specific p53 staining.

We evaluated for a *PDGFRB* N666S mutation in 12/21 cases that had available material for Sanger sequencing (13). Targeted Sanger sequencing of the *PDGFRB* gene was performed as described previously using the following primer sets: Forward (5′-GCCCGCAGCAGTGAGAA-3′) and Reverse (5′-GGTGGGCACTTTCCCTGAG-3′).

## Results and Discussion

While the etiology and cell of origin of Castleman disease neogenesis remain unclear, it is hypothesized that UCD is most likely driven by a neoplastic stromal cell population (10). Recent studies used whole-exome sequencing to demonstrate that the genetic landscape of CD included a frequent *PDGFRB* c.1997 A >G mutation present exclusively in the CD45-negative stromal cells of UCD (13). This finding was reproducible in our index case with an associated spindle cell proliferation, and we further noticed that the allele frequency of the *PDFGRB* c.1997 A >G mutation was higher when enriched for the associated spindle cell proliferation. An analysis of 12 additional cases did not show the presence of this mutation, which may be attributed to the rarity of mutation positive stromal cells present within an extremely lymphoid-rich background and/or the overall low frequency of UCD mutated cases within our cohort (13).

We additionally identified upregulated p53 expression in our index case, which was more concentrated in the area with the predominant spindle cell proliferation, and studied additional cases of UCD, with the hypothesis that p53 may assist in identifying the neoplastic stromal cells of UCD. Twenty-one cases of UCD, hyaline vascular variant showed intermediate to strong nuclear p53 expression within spindle cells associated with this disorder, highlighted by our DIA algorithm in 18/21 cases (~86%), with the remaining 3/21 (~14%) cases (**Figure 2E**) showing rare occasional positive cells based on post-analytical evaluation of the heatmaps (**Figures 2A, B**). Interestingly, one patient had additional regional lymph nodes excised that were not involved by CD and showed reactive follicular hyperplasia. This “control” lymph node from the same patient, and three additional control samples, had no significant p53 expression (**Figures 2C, D**) through our DIA workflow.

Stromal neoplasms, most notably FDC tumors, are known to accompany or develop in the background of Castleman disease. Studies modeling the development of follicular dendritic cells (FDC) show that their progenitor cells can also give rise to other mesenchymal/stromal cell populations of reticular and myoid cells, which have some morphologic, immunophenotypic, and biologic overlap with FDCs (10, 14–16). Interestingly, SMA and caldesmon expression seen on the spindle cell population within our index case shows a spectrum of expression and spatial distribution compatible with the known lineage relationship of FRCs and FDCs, and thus this may be further support for a complex stromal neoplastic cellular origin in UCD (16). Additionally, the presence of p53 expression within stromal cells seen in our UCD cases may reinforce this hypothesis and act as a helpful tool in defining a neoplastic process from a reactive process.

## Data Availability Statement

The raw data supporting the conclusions of this article will be made available by the authors, without undue reservation.

## Ethics Statement

The studies involving human participants were reviewed and approved by the University of California, San Francisco. Written informed consent for participation was not required for this study in accordance with the national legislation and the institutional requirements.

## Author Contributions

Study concept and design: KS, SG, OS, and RSO. Acquisition of data: SG and KS. Analysis and interpretation of data: KS, OS, and RSO. Drafting of the manuscript: KS, OS, and RSO. Critical revision of the manuscript for important intellectual content: KS,SG,JK,AB,CS,SK,EA,RW,OS, and RO. Administrative, technical, or material support, study supervision: RSO. All authors listed have made a substantial, direct, and intellectual contribution to the work and approved it for publication.

## Funding

This study was funded in part by a grant from the Castleman Disease Network to RO, grant number A134657.

## Conflict of Interest

The authors declare that the research was conducted in the absence of any commercial or financial relationships that could be construed as a potential conflict of interest.

## Publisher’s Note

All claims expressed in this article are solely those of the authors and do not necessarily represent those of their affiliated organizations, or those of the publisher, the editors and the reviewers. Any product that may be evaluated in this article, or claim that may be made by its manufacturer, is not guaranteed or endorsed by the publisher.

## Supplementary Material

Supplementary Figure 1 Smooth Muscle Actin (SMA)-positive cells in Unicentric Castleman Disease (UCD). **(A, B)** Representative SMA immunohistochemical stain performed on additional UCD cases show rare circumferential positivity on scattered and grouped non-vasculature cells (magnification x200 and x400).
