# Supplementary figures and images for: Case Report: Castleman Disease With an Associated Stromal Spindle Cell Proliferation, PDGFRB Mutation and p53 Expression: Clonal Origins of a Rare Disease
Source: Front Oncol. 2022 Apr 13;12:857606. doi: 10.3389/fonc.2022.857606 (PMC9043324; doi:10.3389/fonc.2022.857606)

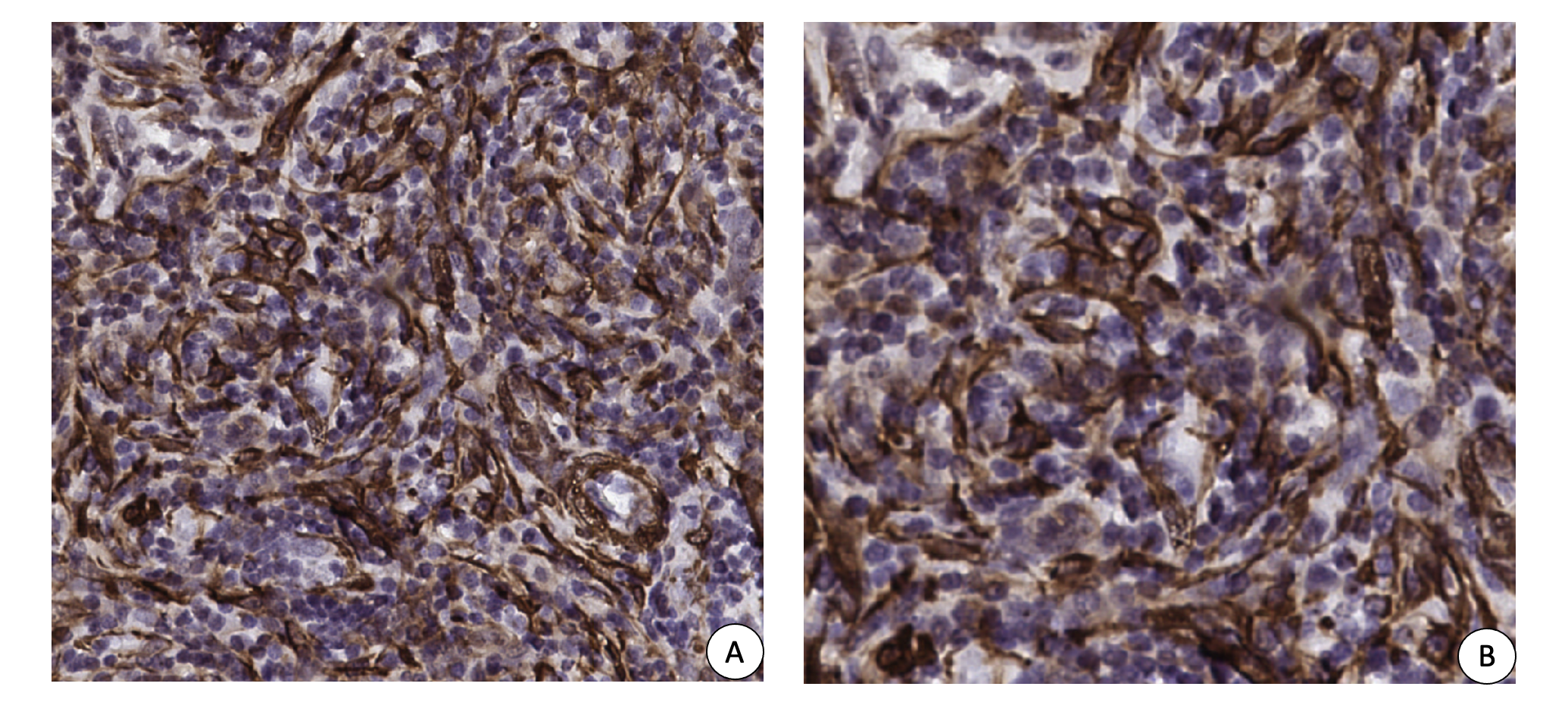

Supplement: Supplementary Figure 1 — Smooth Muscle Actin (SMA)-positive cells in Unicentric Castleman Disease (UCD). (A, B) Representative SMA immunohistochemical stain performed on additional UCD cases show rare circumferential positivity on scattered and grouped non-vasculature cells (magnification x200 and x400). [file DataSheet_1.docx]
